# Formation of Tethered Bilayer Lipid Membranes (tBLMs) Using Plasma Membrane Blebs from Human Lung Cancer Cells

**DOI:** 10.1021/acsomega.6c04781

**Published:** 2026-09-10

**Authors:** Shivabalan Arun Prabha, Martynas Talaikis, Artu̅ras Polita, Vaidas Pudžaitis, Milda Norkienė, Rima Budvytyte, Gintaras Valinčius

**Affiliations:** † Institute of Biochemistry, Life Sciences Centre, 54694 Vilnius University, Vilnius LT-10257, Lithuania; ‡ Department of Organic Chemistry, Center for Physical Sciences and Technology, Vilnius LT-10257, Lithuania; § Institute of Biotechnology, Life Sciences Center, Vilnius University, Vilnius LT-10257, Lithuania

## Abstract

Plasma membrane blebs, or giant plasma membrane vesicles (GPMVs), serve as excellent biomimetics for studying the compositional complexity of native cell membranes. However, current supported membrane models often lack the stability or submembrane reservoir necessary for extended functional studies. This work presents a proof of concept for the formation of tethered bilayer lipid membranes (tBLMs) using blebs derived from human lung cancer cells (A549). We report that successful fusion of blebs into planar tBLMs on gold substrates requires a significantly higher density of tethering anchors (60 mol % WC14) than that required for synthetic liposomes. Surface-enhanced infrared absorption spectroscopy (SEIRAS) analysis indicates that this high anchor density promotes a vertical alignment of the alkyl chains, thereby facilitating the fusion of the complex native membranes. To demonstrate the utility of this platform for pharmacological screening, we investigated the membrane-disrupting mechanism of the anticancer drug edelfosine. Fluorescence lifetime imaging microscopy (FLIM) revealed that edelfosine reduces membrane microviscosity, consistent with a detergent-like permeabilization mechanism. These findings establish bleb-derived tBLMs as a robust platform for applying surface-sensitive analytical techniques to the study of native membrane properties and drug–membrane interactions.

## Graphical abstract

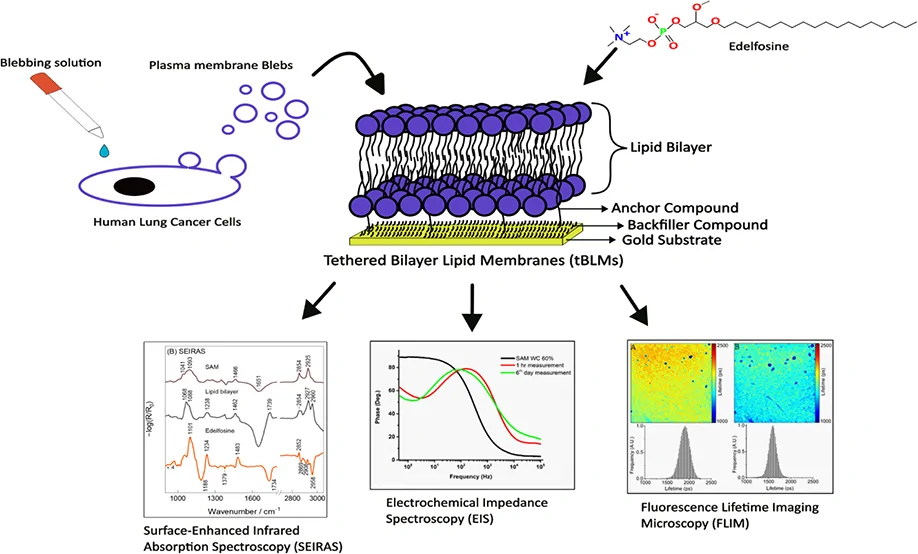

## 1 Introduction

Plasma membranes are crucial in the structural and functional aspects of eukaryotic cells.^1^ The plasma membrane is a vital mediator between extracellular signals and intracellular responses, essential in linking intracellular processes to cell–cell interactions and tissue organization.^2^ Understanding the complexity of the plasma membrane under in vivo conditions can be challenging. One way to overcome this challenge is to isolate the plasma membrane as giant plasma membrane vesicles (GPMVs) or plasma membrane blebs (Blebs).

Blebs are proteoliposomes formed from the plasma membrane that bud off the cell surface.^3^ Blebs are the closest biomimetic models of the plasma membrane in terms of lipid and protein composition and are suitable for a range of analytical techniques.^4,5^ Blebs contain lipids that constitute the plasma membrane, such as phosphatidylcholine (PC), phosphatidylinositol (PI), phosphatidylserine (PS), phosphatidylethanolamine (PE), and phosphatidylethanolamine (PE)-plasmalogen, and are also enriched with sphingomyelin and cholesterol.^6^ As a result, they have been used as tools for both fundamental studies of the plasma membrane and the development of new technologies. Basic studies have used blebs for understanding the lipid packing density, lipid ordered (L_o_) or lipid disordered (L_d_), of the plasma membrane,^7^ to investigate the differences in lipid composition between the plasma membrane and whole cell,^8^ to study the physical properties of the lipid membrane,^9,10^ diffusion patterns of lipids,^11^ and membrane protein interactions.^12,13^ Additionally, blebs have been used for technological applications—Einfalt et al. designed bleb-based artificial cells containing artificial organelles made of nanosized polymerosomes.^14^ Blebs are also reportedly used as drug carriers for targeted delivery of anticancer drugs.^15^

Simplified model membrane systems composed of synthetic lipid species are utilized to investigate the fundamental properties of lipid membranes and the interactions between lipids and membrane proteins. One of the most promising models is tethered bilayer lipid membranes (tBLMs, Figure 1), due to their effectiveness in reconstituting membrane proteins and studying molecular processes in cell membranes.^16,17^ tBLMs are lipid bilayers attached to a solid substrate via a self-assembled monolayer (SAM) composed of anchor and backfiller compounds.^16^ To control the surface density of anchor compounds, they are co-assembled with small backfiller molecules that competitively bind to the substrate, thereby spacing the anchors laterally.^17,18^ Typically, anchor molecules consist of a surface-active thiol group, a hydrophilic poly­(ethylene oxide) spacer, and lipid-like acyl chains that insert into the lipid bilayer and anchor it near the surface. The spacer creates a water-filled space between the solid substrate and the lipid membrane.^19^ This sub-membrane region, approximately 1–2 nm thick, allows the diffusion of ions and functional incorporation of transmembrane proteins.^20^ Additionally, increasing the anchor compound’s length has significantly increased the area of the sub-membrane region, thereby accommodating and stabilizing large membrane proteins. The architecture of tBLMs enables the use of various techniques, such as atomic force microscopy (AFM),^21^ surface plasmon resonance (SPR),^22^ electrochemical impedance spectroscopy (EIS),^23^ fluorescent lifetime imaging microscopy (FLIM),^24^ surface-enhanced infrared absorption spectroscopy (SEIRAS), and others. tBLMs have been used to study the electrical and biophysical properties of membranes formed from lipids in various molar ratios that mimic the mammalian plasma membrane^25,26^ bacterial,^27^ and yeast membranes.^28^ Additionally, tBLMs are used to study the effects of various protein-lipid interactions of pore-forming toxins such as α-hemolysin,^28^ vaginolysin,^29^ and intermedilysin,^30^ as well as the interactions of membrane-modifying molecules such as simvastatin.^31^

**Figure 1:**
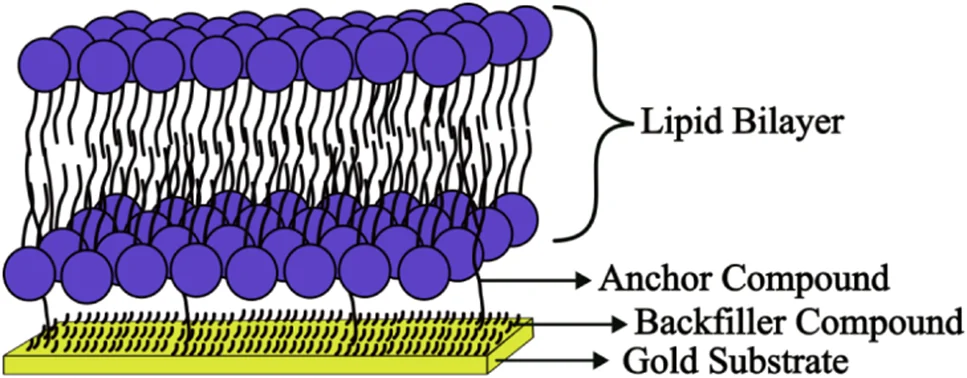
Schematic representation of the architecture of tBLM.

Blebs have previously been fused to form Supported Lipid Bilayers (SLBs) by Susan Daniel’s group.^32^ Although these bleb SLBs have been reported to support the functional incorporation of proteins^33–35^ SLBs pose an inherent disadvantage due to their architecture. In particular, the small distance between the solid surface and the lipid bilayer has been reported to contribute to the instability of the functionalized proteins over time.^36^

Given the advantages of using the tBLM system, we have demonstrated the proof of concept of forming tBLMs using blebs obtained from the A549 lung cancer cell line. We report that the fusion of blebs to form tBLMs requires a higher mol % of the anchor compound, and the resulting tBLMs are electrically stable. Using SEIRAS, we observed changes in structural alignment with increasing anchor compound concentration, resulting in a more vertical alignment with the substrate, thereby providing a possible explanation for the facilitation of bleb fusion to form tBLMs. Next, we used FLIM to examine the interactions between edelfosine, a lipophilic anticancer drug, and bleb-derived tBLMs. Edelfosine has previously been shown to have weak detergent-like effects^37^ and to destabilize model lipid membrane systems at higher concentrations.^38^ Using FLIM, we observed a weak detergent-like effect of edelfosine, as evidenced by changes in microviscosity of bleb-derived tBLMs after edelfosine treatment. Lastly, using SEIRAS, the molecular-level interactions of edelfosine’s detergent-like activity on bleb-derived tBLMs over time were also reported.

Using bleb-derived tBLMs, opens up opportunities to apply various analytical techniques for studying tBLMs and gaining insights into the physical properties of lipid membranes sourced from cells. Additionally, membrane protein functional studies could be possible by overexpressing membrane proteins in cells and obtaining blebs to form tBLMs. Study by Pappa, Anna-Maria et al. 2020 ^33^ reports the presence of functional membrane proteins in blebs that were previously overexpressed in cells. Although blebs have been reported to contain the native membrane proteins.^3,13,32^ This article focuses on the formation of tBLMs from blebs and understanding the interactions of edelfosine on bleb-derived tBLMs. The properties of membrane-bound proteins present in these samples need to be further investigated and are beyond the scope of this study.

## 2 Materials and Methods

### 2.1 Cell Growth and Maintenance

Human lung cancer cells (ATCC: A549) were used for experiments. The cells were maintained at 37 °C with 5% CO_2_ in Dulbecco’s Modified Eagle’s Medium (DMEM) supplemented with 10% FBS, 100 IU/mL penicillin, and 100 μg/mL streptomycin (Thermo Fisher Scientific, US).

### 2.2 Giant Plasma Membrane Vesicles (Blebs) Preparation

Blebs were prepared according to the protocol described in the reference.^4^ Briefly, cells were seeded at a density of ∼10^6^ cells/mL in 3 separate tissue culture dishes (100 × 20 mm, Corning, Merck) and grown for 16–18 h, or until 70% confluency. The cell culture media was removed, and cells were washed three times with PBS (137 mM NaCl, 27 mM KCl, 10 mM Na_2_HPO_4_, 1.8 mM KH_2_PO_4_, pH 7.2) followed by the addition of giant plasma membrane vesicle (GPMV) buffer (10 mM HEPES, 150 mM NaCl, two mM CaCl_2_, pH 7.4). The cells were then incubated with the blebbing chemical agents (25 mM paraformaldehyde (PFA) and 2 mM dithiothreitol (DTT)) dissolved in GPMV buffer. The cells were incubated with these chemical agents for 1 h, after which the bleb suspension was collected and combined from all three culture dishes. This sample was centrifuged at 1500× g for 5 mins at 4 °C to remove cell debris. The supernatant containing the blebs was then dialyzed overnight using a 10 kDa MWCO cassette (Thermo Fisher, US) in GPMV buffer, without the blebbing chemical agents. The dialyzate solution containing the blebs was used for the experiments.

### 2.3 Nanoparticle Tracking Analysis (NTA)

Nanoparticle tracking analysis (Nanosight, Malvern, UK) was used to identify the concentration of the dialyzed blebs solution.

### 2.4 Preparation of Large Unilamellar Vesicles (LUVs)

DOPC and cholesterol solutions in chloroform (10 mM) were mixed in a molar ratio of 60:40. Then, chloroform was evaporated under a N_2_ stream. The lipid film was then hydrated by adding PBS (137 mM NaCl, 27 mM KCl, 10 mM Na_2_HPO_4_, 1.8 mM KH_2_PO_4_, pH 7.2) and sonicated for 60 min with occasional vortexing until the lipid film was completely dissolved. The preparation was then extruded 21 times through a 200 nm polycarbonate membrane (Avanti Polar Lipids, Alabaster, US) using glass syringes.

### 2.5 tBLM Preparation

Glass substrates were coated with a 10 nm Cr adhesion layer and a 100 nm Au layer using a magnetron sputtering system (Kurt J. Lesker PVD 75, US). The substrates were then incubated for 12 h at room temperature with 0.05 mM thiol mixture in isopropanol to form SAMs. The thiol mixture consisted of 30:70, 40:60, 50:50, or 60:40 (mol %:mol %) ratios of anchor thiol WC14 (20-tetradecyloxy-3,6,9,12,15,18,22-heptaoxahexatricontane-1-thiol; synthesized in-house^39^)­(Figure S1) and backfiller β-mercaptoethanol (ME; Sigma-Aldrich, USA). After incubation, substrates were rinsed with isopropanol, dried under a nitrogen stream, and assembled into an electrochemical cell. Vesicles were added to the cell for tBLM formation. The cell consisted of 14 independent 250 μL wells, each with a surface area of 0.16 cm^2^.

### 2.6 Fluorescence Lifetime Imaging Microscopy (FLIM) and Fluorescence Microscopy

FLIM analysis was performed on cells seeded at ∼10,000 cells/mL in a 35 mm high μ-Dish (Ibidi GmbH, Germany) and cultured for 16–18 h. Cells were stained with BODIPY-PM (1 μM aqueous solution) for 2 min prior to imaging.^24^ For fluorescence microscopy, cells grown in the μ-Dish were washed three times with PBS and stained with Fast DiO (Thermo Fisher Scientific, US) for 60 min, after which the cells were subjected to blebbing. The blebs formed in suspension were collected and transferred into a 24-well plate. Fluorescence imaging of cells and blebs was performed with a light microscope (Olympus IX51, Japan), and bleb diameters were measured in ImageJ. For FLIM measurements, bleb-derived tBLMs were formed by incubating 1 mL of bleb suspension for 1 h on gold-coated mica substrates functionalized with a WC14:ME SAM (60:40 mol/mol). The membranes were gently washed 3–5 times with GPMV buffer, followed by a 3 min incubation with an aqueous solution of BODIPY-PM (1 μM). Excess dye was washed away with PBS. After acquiring initial images, 100 μM edelfosine was added directly to the tBLMs for a 5 min incubation.

### 2.7 Contact Angle Measurements

The hydrophobicity of different SAMs was measured using the contact angle of a sessile water drop of about 1–2 μL. The contact angle was measured using the droplet shape analyzer DSA 100 (Kruss GmbH Hamburg, DE) in a chamber at 20 °C, with at least three reproducible measurements on each surface.

### 2.8 Electrochemical Impedance Spectroscopy (EIS)

EIS was measured using an electrochemical workstation Palm Sense 4 (Palm Sense B.V., The Netherlands). The impedance spectra were obtained in potentiostatic mode with 10 mV alternating current at 0 V bias versus the Ag/AgCl reference electrode (Sigma-Aldrich, USA) in aerated solutions. The frequency range was from 0.5 Hz to 100 kHz, with 10 logarithmically distributed measurement points per decade. The impedance data were normalized to the surface area.

### 2.9 Infrared (FTIR) and Surface-Enhanced Infrared Absorption Spectroscopy (SEIRAS)

FTIR spectra were acquired using an Alpha spectrometer (Bruker, Germany) equipped with a diamond ATR accessory. Data were collected over 100 scans at a resolution of 4 cm^–1^.

SEIRAS experiments were performed on a Vertex 80v spectrometer (Bruker, Germany) equipped with a liquid-nitrogen-cooled MCT detector, a VeeMax III accessory, and a Jackfish J1F electrochemical cell (Pike Technologies, USA) at a 50° angle of incidence. Spectra were collected at 4 cm^–1^ resolution with a 2 mm aperture, co-adding 50 samples and 100 background scans. A polarizer perpendicular to the wafer surface was employed throughout. The substrate preparation for SEIRAS is reported elsewhere.^40^ Briefly, a Si wafer (IRUBIS GmbH, Germany) was polished using a water-based diamond suspension with 0.25 μm particles for 5 min. Then, the wafer was cleaned with ultrasound in acetone (2 times) and water (2 times), each for 10 min. The wafer surface was incubated with 2 wt % HF for at least 4 min, rinsed with water, and coated with Au plating mixture for 4 min at room temperature. The Au plating mixture was prepared by combining equal volumes of four distinct aqueous solutions immediately before use: (1) 0.15 M Na_2_SO_3_, 0.05 M Na_2_S_2_O_3_, 0.05 M NH_4_Cl, (2) NH_4_F (20 wt %), (3) HF (2 wt %), and (4) NaAuCl_4_ (0.03 M). To increase the mechanical resistance of the Au film, the first Au layer was removed with aqua regia and coated with an Au plating mixture for an additional 4 min.^41^ Electropolishing was performed in deoxygenized sodium acetate solution (0.1 M, pH 5.8) using PGSTAT101 potentiostat (Methrom, USA). The initial potential range was 0.0–0.3 V at 0.02 V/s. After every three scans, the upper limit was increased by 0.1 V until reaching approximately 1.0 V, where gold oxidation occurs.

## 3 Results and Discussion

### 3.1 Production of Blebs

The human non-small cell lung cancer cell line A549 (Figure 2A) is a widely used model system for studying the biochemistry of cancer cells and the interaction of various drug compounds screened for cancer growth inhibition. Blebs (white arrows, Figure 2B) can be produced by chemical induction with chemical agents such as N-ethyl maleimide (NEM)^42^ or the combination of para-formaldehyde (PFA) and dithiothreitol (DTT).^4^ The PFA/DTT combination is the most commonly used chemical agent for producing blebs. Previous studies have confirmed three key properties of these blebs: (1) their ability to form bilayers on solid supports,^34^ (2) the preservation of coexisting liquid-ordered and liquid-disordered phases,^9,43^ and (3) measurable lipid diffusion within the resulting supported membranes.^11^

**Figure 2:**
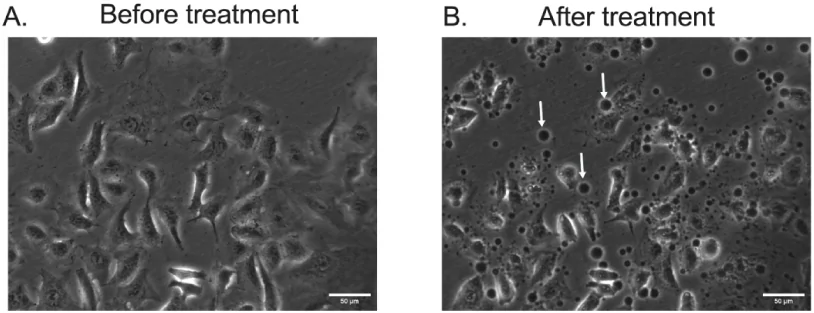
Bright-field microscopy images of A549 cells (A) before and (B) after treatment with blebbing chemical agents (PFA/DTT) to obtain blebs (white arrows).

In this study, blebs were generated using the standard PFA/DTT protocol. Across four independent trials, the average concentration was 1.83 (±0.39) × 10^^9^ particles/mL. The yield of GPMVs is known to depend heavily on the cell type; for instance, Dey et al.^44^ demonstrated that A549 cells produce significantly higher particle counts than Vero or HEK293T cells under identical induction conditions. Our isolated blebs ranged in size from 2 to 25 μm, with a mean diameter of 9.37 ± 5.27 μm as determined by fluorescence microscopy (Figure S2C). Similar size distributions have been reported by Einfalt et al.^14^ for blebs derived from HepG2 liver carcinoma cells using this isolation protocol.

To understand the changes in the microviscosity of the plasma membrane of cells during the process of blebbing, we performed fluorescence lifetime imaging microscopy (FLIM) in combination with viscosity-sensitive probe BODIPY-PM,^24^ which accumulates explicitly in the outer leaflet of plasma membranes and displays fluorescence lifetime sensitivity to environmental microviscosity, with high viscosities yielding high fluorescence lifetimes, and vice versa.

To further characterize membrane-derived vesicles formed following the treatment with blebbing chemical agents, FLIM measurements of BODIPY-PM were performed in live A549 cells (Figure 3). This information would provide a reference for evaluating the extent to which the bleb-derived biomimetic platform would preserve the native membrane characteristics.

**Figure 3:**
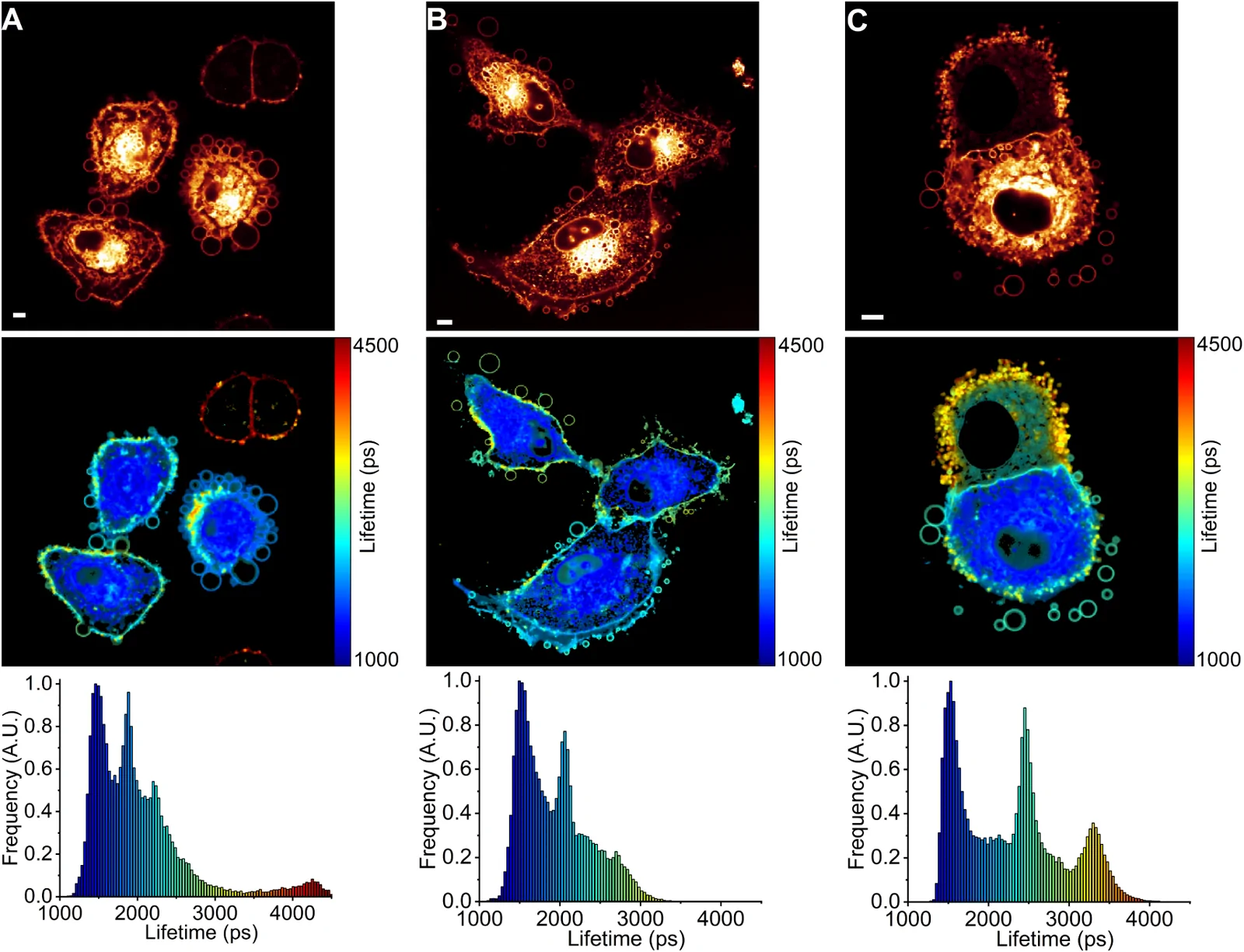
FLIM of BODIPY-PM in live A549 cells following treatment with blebbing chemical agents. Fluorescence intensity images (top), corresponding fluorescence lifetime maps (middle), and fluorescence lifetime histograms (bottom). (A) Cell exhibiting a largely intact plasma membrane and a fluorescence lifetime of approximately 4500 ps. (B) Cell producing membrane-derived vesicles with heterogeneous fluorescence lifetimes. (C) Cell producing relatively homogeneous membrane-derived vesicles with elevated fluorescence lifetimes. Scale bars: 5 μm.

Untreated regions of the plasma membrane and cells that did not visibly respond to treatment exhibited fluorescence lifetimes of approximately 4500 ps (Figure 3A). In contrast, membrane-derived vesicles generated after treatment with blebbing agents displayed substantially shorter fluorescence lifetimes, indicating altered membrane microenvironments relative to the intact plasma membrane (Figure 3). The observed fluorescence lifetimes varied considerably between vesicles, ranging from approximately 1250 to 3500 ps. In addition, some cells produced vesicles with a broad distribution of fluorescence lifetimes, whereas others generated vesicles with more homogeneous lifetime values (Figure 3B,C). These observations indicate considerable heterogeneity among membrane-derived vesicles formed during the treatment with blebbing chemical agents and suggest that their membrane properties differ from those of the native plasma membrane.

### 3.2 tBLM Formation by Electrochemical Impedance Spectroscopy

Using electrochemical impedance spectroscopy, we monitored the formation of tBLMs from both native blebs and synthetic DOPC/Cholesterol vesicles to identify differences in membrane resistance and capacitance. EIS confirmed membrane formation via characteristic Cole–Cole features^45^ and a reduction in surface capacitance from 10 μF/cm^2^ (SAM) to 1 μF/cm^2^ (tBLM). The diameter of the semicircular feature of EIS spectra is proportional to the capacitance of tBLM.^39^ The Bode plots represent features that reflect the presence of defects on tBLMs; the appearance of a frequency minimum (*F*_min_) in the sample spectra compared to the SAM spectra indicates membrane formation and is also used to assess the admittance of the bilayer.^39,46^ In general, tBLM studies that employ Multilamellar Vesicle (MLV) fusion use ∼30 mol % of the anchor compound for bilayer formation.^47,48^ Initial experiments focused on bleb fusion using a lower SAM concentration (WC 30 mol %). This resulted in no membrane formation, with the bleb sample spectra showing semicircular diameters similar to those of the SAM spectra, as observed in the Cole–Cole plot (Figure S3A).

Additionally, the absence of *F*_min_ in the Bode plot (Figure S3B) indicates no fusion of blebs. Since we observed that the fusion characteristics of the bleb samples differ from those of commonly used MLVs, we optimized the underlying SAM layer by increasing the concentration of the anchor molecule to make the surface more hydrophobic, thereby facilitating the fusion of blebs on the SAM to form tBLMs. The phenomenon of increasing surface hydrophobicity to facilitate fusion has previously been studied in synthetic unilamellar vesicles.^49,50^ It was shown that when large unilamellar vesicles (LUVs) adsorb onto the hydrophobic SAM surface, mechanical stress is exerted on the vesicle surface, leading to pore formation, followed by lipid unraveling and spreading across the SAM layer. The increase in surface hydrophobicity upon increasing the anchor compound concentration was measured using contact angle experiments (Figure S4), and the results were consistent with previous studies.^39,51^ EIS data revealed that LUV and bleb did not fuse on 40 mol % of the anchor compound SAM (Figure S3C,D). Increasing the WC anchor density to 50 mol % successfully induced LUV fusion, evidenced by a reduction in the Cole–Cole semi-circle diameter and the emergence of a distinct c in the Bode plots (Figure S3E,F). In contrast, this surface composition proved insufficient to initiate tBLM formation in the bleb samples.

At 60 mol % WC, both LUVs and blebs formed bilayers, evidenced by a collapsed semi-circle in the Cole–Cole plots (Figure 4A, C) and the appearance of a distinct *F*_min_ in the Bode plots (Figure 4B, D) relative to the SAM controls. It is important to note that the Bode plots of 1 h and 1 day measurements (Figure 4B, red and green squares) of the LUV bilayer showed high resistances, and the *F*_min_ could not be visualized at these frequencies; therefore, it would be necessary to measure the membrane at lower frequencies. Capacitance analysis reveals that bleb-derived tBLMs have higher capacitance values of 0.87 μF/cm^2^ compared to the capacitances of LUVs tBLMs, which reached 0.78 μF/cm^2^ and were stable over time (Figure 4E and Table S1). The significant cholesterol content in LUVs reduces membrane capacitance by increasing membrane thickness and decreasing the dielectric constant.^52,53^ The whole Cole–Cole plots exhibit a typical “two semi-circle” shape, a characteristic of disrupted tBLM integrity (Figure 4A, B). Mathematical analysis shows that a well-expressed secondary semicircle in Cole–Cole EIS plots arises from a greater number of natural defects in the initial membrane.^47,48^

**Figure 4:**
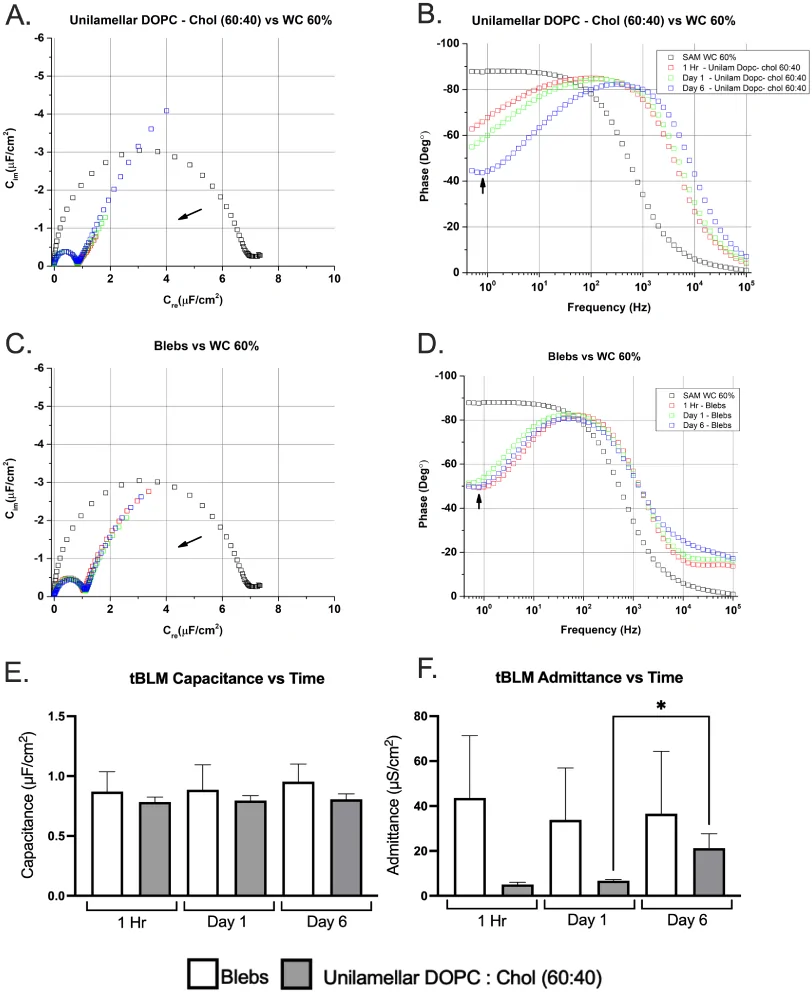
Comparison of the EIS data showing tBLM formation of unilamellar vesicles (A, B) and blebs (C, D) on WC14:ME (60:40 mol %) SAM. The formation of tBLMs can be inferred in the Cole–Cole plot by the decrease in the diameter semi-circular feature (arrows) between the SAM spectrum of the unilamellar vesicles (A) and blebs (C). The formation of tBLMs can also be inferred from the appearance of *F*_min_ (arrows) in the Bode plot of unilamellar vesicles (B) and blebs (D) compared to the SAM: the capacitance (E) and the admittance changes (F) of tBLMs over time.

Admittance analysis of EIS spectra indicates that the bleb-derived tBLM has higher admittance values of 43 μS/cm^2^ as compared to LUV membranes having an admittance of 5.07 μS/cm^2^ (Figure 4F, Table S1). Bleb-derived tBLM can induce phase separation, with raft and non-raft regions exhibiting distinct physical properties and creating membrane packing defects. These defects may alter ion permeability or provide sites for ion flux.^54^ Moreover, since both blebs and blebbing cell plasma membranes exhibit decreased fluorescence lifetimes of BODIPY-PM, and thus lower microviscosities, compared to non-blebbing cells, the lipid composition of tBLMs prepared from blebs might reflect the plasma membrane’s state during blebbing process.

### 3.3 Anchor Compound Structural Changes by SEIRAS

The anchoring monolayer used here, WC14 co-assembled with ME, is a well-characterized tethering system whose molecular-scale architecture has been resolved by neutron reflectometry (NR), providing a direct depth profile of the SAM and the completed tBLM normal to the gold surface.^39^ Pure WC14 SAMs form a tightly packed, strongly hydrophobic alkyl layer over a largely dehydrated heptaethylene oxide spacer (submembrane thickness d_sub_ ≈ 16.7 Å, exchangeable-solvent fraction ≈ 5 vol %). Co-adsorption of the shorter ME backfiller lowers the WC14 surface density and opens a hydrated submembrane reservoir, with the exchangeable-solvent fraction rising to 47–60 vol % at a 50:50 solution ratio and to 60–75 vol % at 30:70, while d_sub_ contracts to ≈13 Å.^39^ A steplike rise in SAM capacitance below 70 mol % WC14 marks the composition at which the anchors cease to form a continuous hydrophobic film.^39^ These NR results directly establish the sub-nanometer interfacial architecture and submembrane hydration; the SEIRAS measurements below complement them by resolving the in situ conformational state of the anchor chains under aqueous bleb-fusion conditions. Since the 60:40 WC:ME composition gave better fusion of Blebs (Figure 4D) along with its similar SAM electrical properties to that of the 30:70 WC:ME composition (Figure 4C and Figure S3A), we were motivated to probe these two compositions for structural characterization.

To elucidate the molecular-level structural changes of the anchoring monolayers and their influence on bleb fusion under in situ conditions, we performed SEIRAS measurements of SAMs composed in 30:70 and 60:40 molar ratios of WC14:ME (Figure 5A). The vibrational bands clearly show the presence of SAM on the gold substrate surface, evidenced by the intense broad mode at 1103–1113 cm^–1^ and a higher energy shoulder, which are associated with the characteristic asymmetric stretching motion of the poly­(ethylene glycol) (PEG) segment of WC14, ν_as_(C–O–C).^55–63^ The wagging motion of CH_2_ of the PEG in trans conformation at 1325 cm^–1^ is very weak, while the gauche wagging band could be found at 1350 cm^–1^.^57^ The ∼1110 and 1350 cm^–1^ peak positions are consistent with a helical conformation of the PEG chain, while the ones of the shoulder at higher frequencies (1140–1152 cm^–1^) are associated with the amorphous states PEG.^60–62^ The mode at 1466 cm^–1^ belongs to the scissoring of methylene groups, and the 2855 and 2927 cm^–1^ to the symmetric and asymmetric stretchings, ν_sym_(CH_2_) and ν_as_(CH_2_), respectively.^55–58^ The weak features near 2874 and 2958 cm^–1^ appear due to the ν_sym_(CH_3_) and ν_as_(CH_3_) motion of WC14 chains-terminal methyl groups.

**Figure 5:**
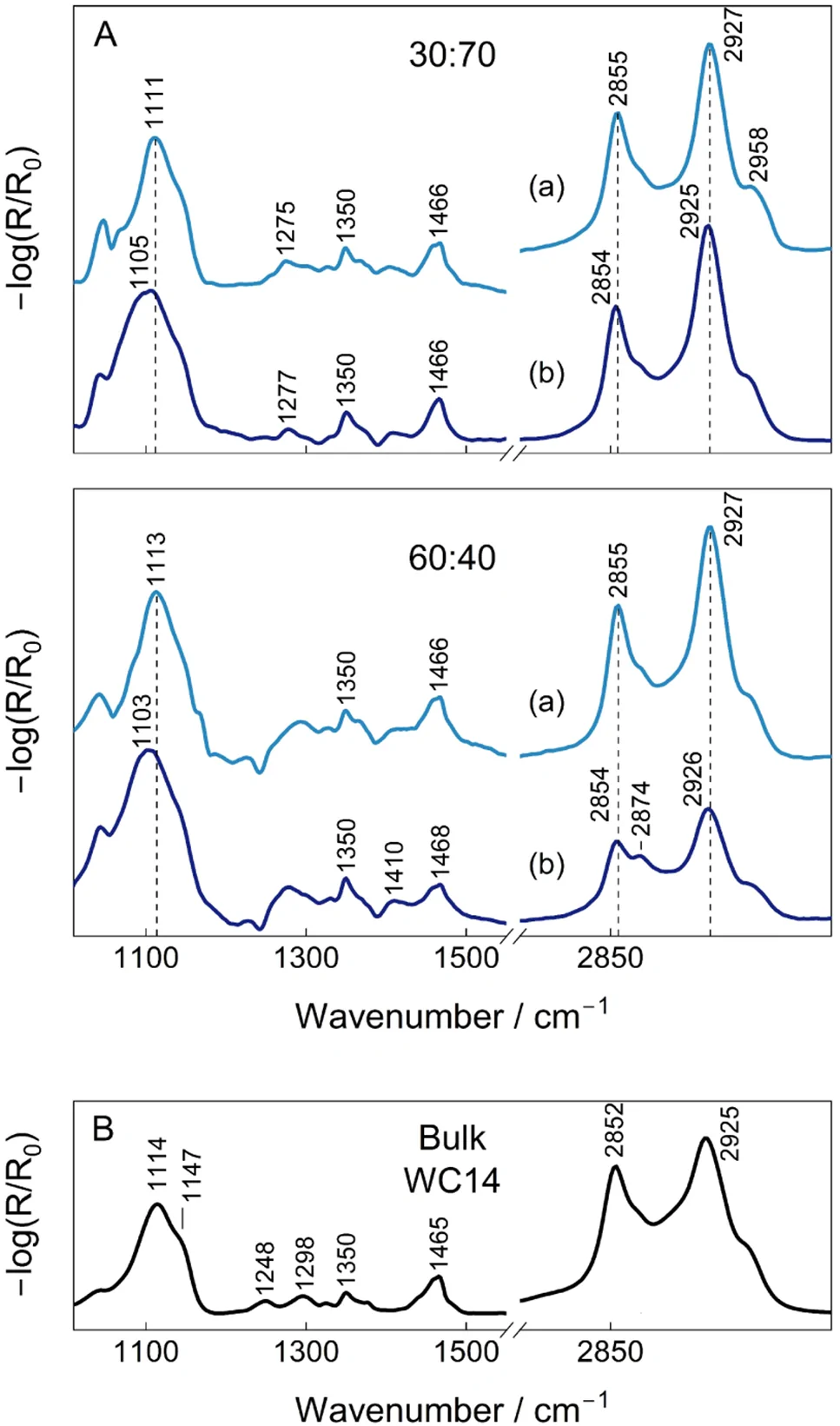
(A) SEIRAS spectra of self-assembled monolayers composed of 30:70 and 60:40 ratios of WC14:ME in ethanol solution after 1 h incubation (a) and immediately after exchanging with HEPES solution pH 7.4 (b). The ethanol and buffer spectra are subtracted. (B) The infrared absorption spectrum in the transmission mode of WC14 deposited on a CaF_2_ substrate.

The rearrangement of the anchoring layer molecules is evident following the exchange of the ethanolic incubation solution with a HEPES buffer pH 7.4. For both SAM compositions, the ν_as_(C–O–C) mode in the ethanol spectra appears close to what was found in a spectrum of weakly hydrogen-bonded bulk WC14 (Figure 5B). Shifts to lower wavenumbers by 6–10 cm^–1^ upon exposure to an aqueous buffer solution indicate increased hydrogen-bonding strength at the PEG moiety and are consistent with the literature.^63^ Similarly, the ν_sym_(CH_2_) and ν_as_(CH_2_) modes move to lower frequencies by approximately 1.1–1.5 cm^–1^. Despite the small scale, changes in C–H stretching modes are highly reproducible and are linked to increased packing of hydrophobic alkane chains upon exposure to a higher-polarity solvent.^56^

Uniquely to the 60:40 SAM, infrared absorption drastically decreases in the C–H stretching region after ethanol/HEPES exchange. In SEIRAS, vibrational modes, whose transition dipole moments are perpendicular to the metal surface, are substantially enhanced, while those aligned parallel exhibit marked attenuation.^64^ The observed twofold reduction in ν_sym_(CH_2_) and ν_as_(CH_2_) intensities indicates a reorientation of the methylene transition dipole moments parallel to the substrate surface, a phenomenon consistent with the vertical ordering of WC14 alkane chains in aqueous solution. On the contrary, for the 30:70 composition, the chains remain primarily unaffected by solvent exchange. Also, no considerable orientational changes were detected for PEG chains in either of the compositions. According to the contact angle measurements of the sessile drop in Figure S4, surface hydrophobicity increased with higher anchor concentration, governed by the reorientation of alkyl chains in contact with water.

### 3.4 Edelfosine Interaction with Bleb-Derived tBLMs

To demonstrate the utility of bleb-derived tBLMs in characterizing ligand-membrane interactions, we employed FLIM to assess the effects of edelfosine (Figure S5). This anticancer agent was selected for its established capacity to compromise membrane integrity and induce permeabilization in cellular systems. Edelfosine has been reported to exhibit weak detergent-like activity and destabilize model lipid membrane systems at higher concentrations.^37,38^ FLIM measurements of prepared bleb-derived tBLM indicate that the average fluorescence lifetime of BODIPY-PM was 1685 ps, which corresponds to a viscosity of 89 cP in the calibration mixtures of methanol and glycerol (Figure 6A).^24^ Upon the addition of 100 μM edelfosine, the average fluorescence lifetime of BODIPY-PM decreased from 1685 to 1445 ps within 5 min. This shift corresponds to a microviscosity of 52 cP, indicating a reduction in the lipid bilayer’s microviscosity (Figure 6B). We hypothesize that this decrease in microviscosity results from edelfosine’s detergent-like action,^37,65^ which may lead to lipid depletion, reduced bilayer ordering, and increased probe mobility in the local environment.

**Figure 6:**
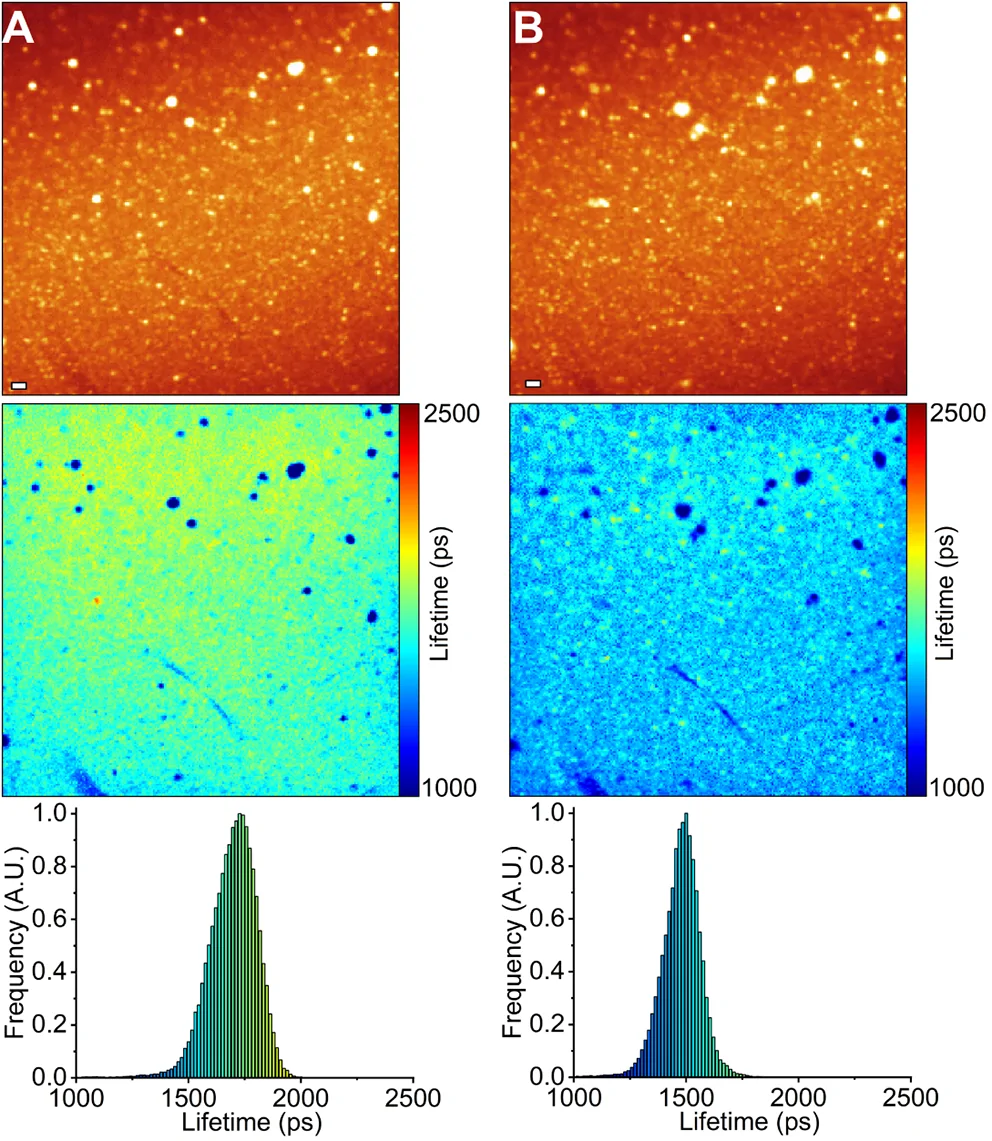
FLIM of BODIPY-PM in GPMV-derived tBLMs. Fluorescence intensity images (top), corresponding fluorescence lifetime maps (middle), and fluorescence lifetime histograms (bottom). (A) Initial tBLMs and (B) tBLMs treated with edelfosine (100 μM) for 5 min. Scale bars: 1 μm.

SEIRAS measurements were conducted to probe the molecular mechanism of the edelfosine–membrane interaction. The reference FTIR spectrum of solution edelfosine in Figure 7A (a) is dominated by vibrational bands at 1087 and 1223 cm^–1^ originating from the phosphate group, specifically the ν_s_(PO_2_) and ν_as_(PO_2_) modes. SEIRAS spectra show a sequential formation of SAM (b), bleb-derived tBLM (c), and tBLM after edelfosine injection (d). The bleb-derived tBLM spectrum is characterized by amide II (1548 cm^–1^) and amide I (1664 cm^–1^) features, which are characteristic of membrane proteins. Upon edelfosine injection, a progressive spectral evolution occurs, and after 1 h, the spectrum becomes completely dominated by the drug’s vibrational features (d). The ν_s_(PO_2_) and ν_as_(PO_2_) shift to higher frequencies upon interaction with tBLM compared to FTIR. Both of these vibrations are sensitive to the phosphate group’s hydration level and may shift as much as 15–30 cm^–1^ (ν_as_) and 5 cm^–1^ (ν_s_) depending on changes in the environment’s polarity.^66,67^ Consequently, the observed 4–5 cm^–1^ wavenumber shift to higher frequencies indicates decreased hydration of phosphate groups, likely reflecting the drug’s insertion into the lipid bilayer’s lower-polarity environment. Overall, the wavenumber shift reaches maximum values at 90 s (1092 and 1229 cm^–1^), and then is followed by a slight but steady decrease in wavenumber (Figure 7B, black squares for ν_as_(PO_2_) mode). These data are consistent with a detergent-like mechanism in which edelfosine compromises the tBLM integrity, leading to significant shifts in the local polarity surrounding the lipid and drug molecules. The ν­(CH_2_) intensity at 2925 cm^–1^ is initially negative, indicating a net decrease relative to the tBLM reference, and remains so for approximately the first 3 min following edelfosine injection (Figure 7B, red diamonds). In SEIRAS, vibrational modes whose transition dipole moments are oriented parallel to the surface are attenuated.^64^ The observed initial decrease in intensity therefore indicates that edelfosine insertion promotes a more vertical ordering of the membrane alkyl chains, consistent with a condensing effect at early interaction times. After approximately 3 min, the intensity increases above the baseline, reflecting a loss of this ordered packing, likely due to progressive membrane disruption and lipid extraction by edelfosine’s detergent-like action, as corroborated by the simultaneous evolution of the ν_as_(PO_2_) frequency (Figure 7B, black squares).

**Figure 7:**
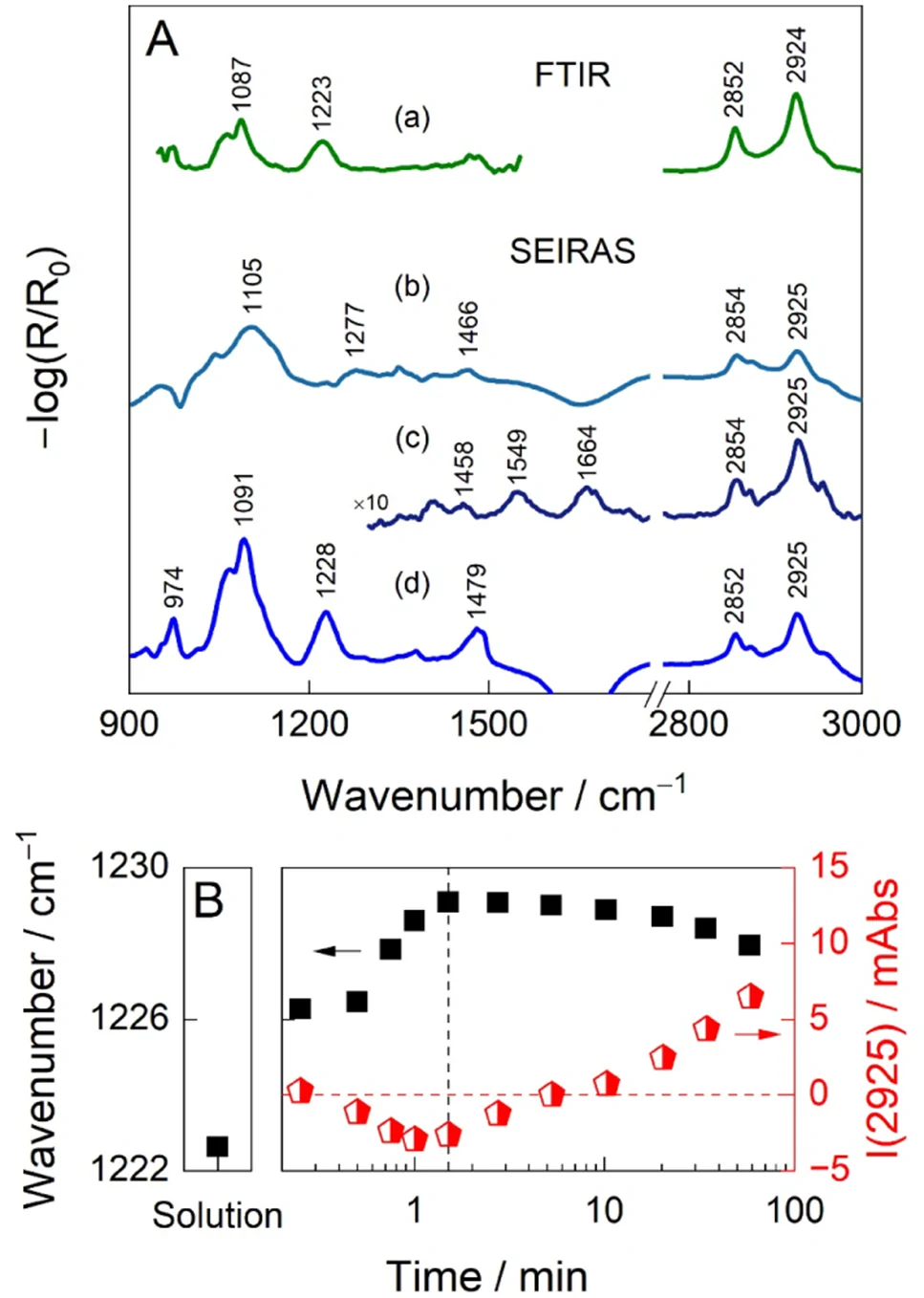
(A) FTIR spectrum of water-dissolved edelfosine (a) and SEIRAS spectra of the 30:70 WC14:ME SAM (b), the bleb-derived tBLM (c), and the bleb-derived tBLM 60 min after injection of 0.1 mM edelfosine (d). All SEIRAS spectra (b–d) were recorded in HEPES buffer (pH 7.4). (B) The time-dependent evolution of wavenumbers of asymmetric stretching of phosphate group ν_as_(PO_2_) (black squares) and the intensity of asymmetric CH_2_ stretching ν­(CH_2_) at 2925 cm^–1^.

To establish a baseline for the native bleb-derived tBLMs, we first characterized a synthetic model membrane composed of DOPC and cholesterol (60:40 mol %). This protein-free reference facilitates the isolation of lipid-specific vibrational signatures from the complex composite signals typical of native membranes. As shown in Figure S6, the SEIRAS spectrum of the synthetic tBLM is dominated by characteristic lipid features, including the methylene stretching vibrations ν­(CH_2_) at 2850–2925 cm^–1^ and a distinct ester carbonyl band ν­(C=O) at 1739 cm^–1^. Crucially, the synthetic model lacks the amide I and amide II bands, confirming that the protein signals observed in the bleb-derived tBLMs (Figure 7) are unique to the native cellular material.

Upon the injection of edelfosine into the synthetic system, we observed a progressive decrease in the intensity of the ester carbonyl ν­(C=O) at 1734 cm^–1^ and the C–O ester bands 1188 cm^–1^ over 60 min (Figure S7).^68^ The negative sign of these bands in the difference spectra suggests a loss of lipid mass from the tethered architecture, consistent with a detergent-like mechanism where edelfosine actively extracts DOPC molecules from the bilayer. Furthermore, the spectral evolution points to a structural rearrangement of the remaining lipid phase, potentially indicating a reorientation of the terminal methyl groups (2869 and 2958 cm^–1^) toward a more vertical alignment relative to the substrate surface.

SEIRAS data demonstrate that the bleb-derived tBLMs retain the compositional complexity of the source plasma membrane, evidenced by the persistence of protein-specific vibrational modes, while exhibiting the physical responsiveness expected of a fluid bilayer. The capacity to resolve synchronous lipid and protein removal validates the platform’s utility for monitoring gross membrane reorganization and lysis events.

## 4 Conclusions

We report the formation of planar, tethered bilayer lipid membranes (tBLMs) directly derived from plasma membrane blebs of human lung cancer cells (A549). While blebs have previously been utilized to generate supported lipid bilayers (SLBs),^32^ such models possess limited stability in addition to having a thin hydration layer in their submembrane region.^69^ By reconstituting native blebs into a tBLM architecture, we successfully established a biomimetic platform that exhibited long-term electrical stability, as demonstrated by EIS measurements, while preserving native membrane characteristics, as confirmed by FLIM. Additionally, a previous NR study of mixed SAM containing WC14 and ME backfiller in the solution ratio of 50:50 contained 40–60% of hydrated submembrane region.^39^ Unlike synthetic liposomal formulations, which fuse readily at lower densities, native blebs required a high anchor concentration (60 mol % WC14). Surface-enhanced infrared absorption spectroscopy (SEIRAS) analysis suggests that this high density promotes a vertical conformational alignment of the anchor alkyl chains.

We have also tested the effects of the lipophilic anticancer drug edelfosine. FLIM analysis revealed a significant reduction in membrane microviscosity, attributed to lipid extraction, while time-resolved SEIRAS confirmed the drug’s detergent-like kinetics on the bleb-derived architecture. To our knowledge, this is the first report of tBLMs fabricated directly from native plasma membrane blebs. By preserving the compositional complexity of the source tissue, this platform provides a superior biomimetic interface for investigating lipid-protein interactions and screening membrane-targeting therapeutics.

## Supplementary Material



## Data Availability

Data will be made available upon request.

## Notes

Figure S1: Structure of WC14 [20-tetradecyloxy-3,6,9,12,15,18,22-heptaoxatricontane-1-thiol]; Figure S2: Fluorescent microscopy of A549 cells, Blebs and their size distribution plot; Figure S3: Electrochemical impedance spectra of the blebs and unilamellar vesicles on increasing concentrations of the anchor compounds that constitute the SAM layer; Figure S4: Contact angle measurement plot showing the increasing contact angle values for increasing WC molar fractions; Table S1: Temporal evolution of the specific capacitance (μF cm^–2^) and admittance (μS cm^–2^) for tBLMs derived from blebs and unilamellar vesicles over 6 days; Figure S5: Structure of the anti cancer agent Edelfosine; Figure S6: Transmission FTIR spectra of bulk lipids and SEIRAS spectra of the SAM at different anchor densities; Figure S7: Time-resolved SEIRAS difference spectra of the synthetic DOPC/cholesterol tBLM and the bleb-derived tBLM upon interaction with edelfosine (PDF)
