# Supplementary material for: Formation of Tethered Bilayer Lipid Membranes (tBLMs) Using Plasma Membrane Blebs from Human Lung Cancer Cells
Source: ACS Omega. 2026 Sep 10;11(37):55665–74. doi: 10.1021/acsomega.6c04781 (PMC13613449; doi:10.1021/acsomega.6c04781)
Supplement: Supplementary file 1 [file ao6c04781_si_001.pdf]

Supporting information

Formation of Tethered Bilayer Lipid Membranes (tBLMs) Using Plasma Membrane Blebs of Human Lung Cancer Cells

Shivabalan Arun Prabha[1], Martynas Talaikis[1], Artūras Polita[1], Vaidas Pudžaitis[2], Milda Norkienė[3], Rima Budvytyte[1] and Gintaras Valinčius[1]

[1]Institute of Biochemistry, Life Sciences Centre, Vilnius University, Vilnius, LT-10257, Lithuania.

[2] Department of Organic Chemistry, Center for Physical Sciences and Technology, Vilnius, LT-10257, Lithuania.

[3]Institute of Biotechnology, Life Sciences Center, Vilnius University, Vilnius, LT-10257, Lithuania.

WC14

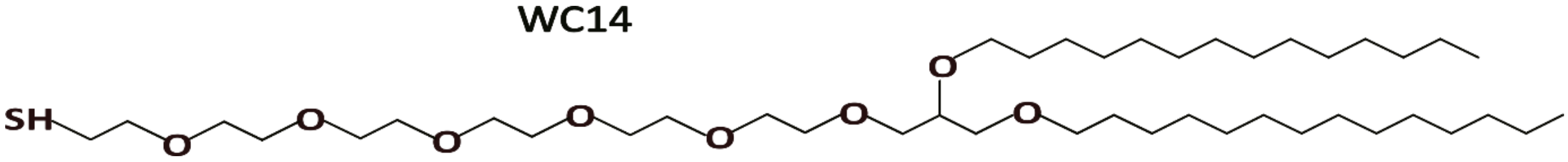

Figure S1. Structure of WC14 [20-tetradecyloxy-3,6,9,12,15,18,22-heptaoxatricontane-1-thiol].

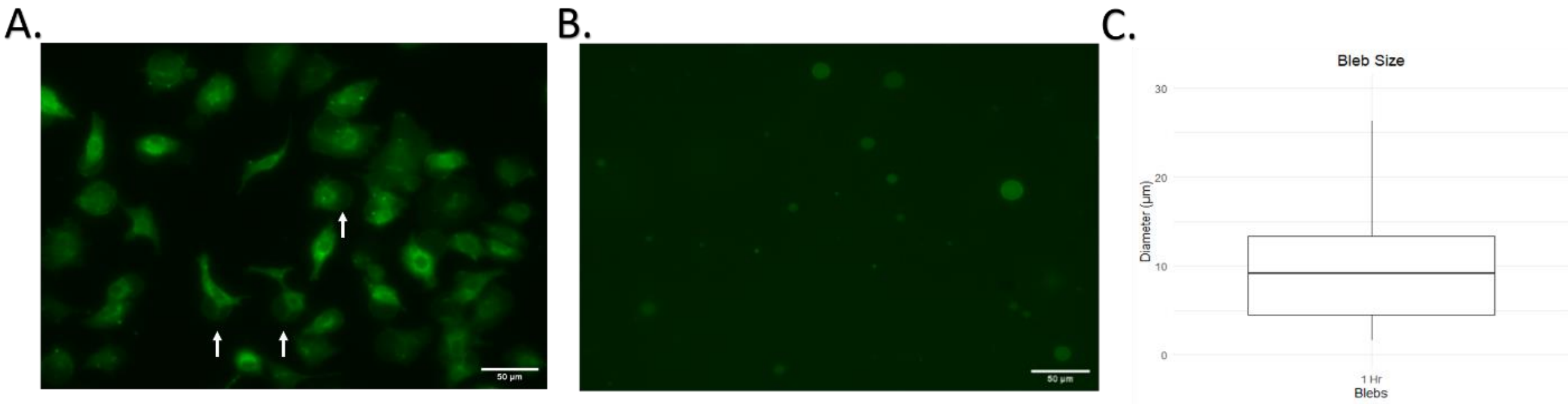

Figure S2. Fluorescent microscopy of A. A549 cells producing Blebs(white arrows), B. The Blebs containing the fluorescent dye and C. The plot showing the size heterogeneity of the obtained Blebs (n=20 images).

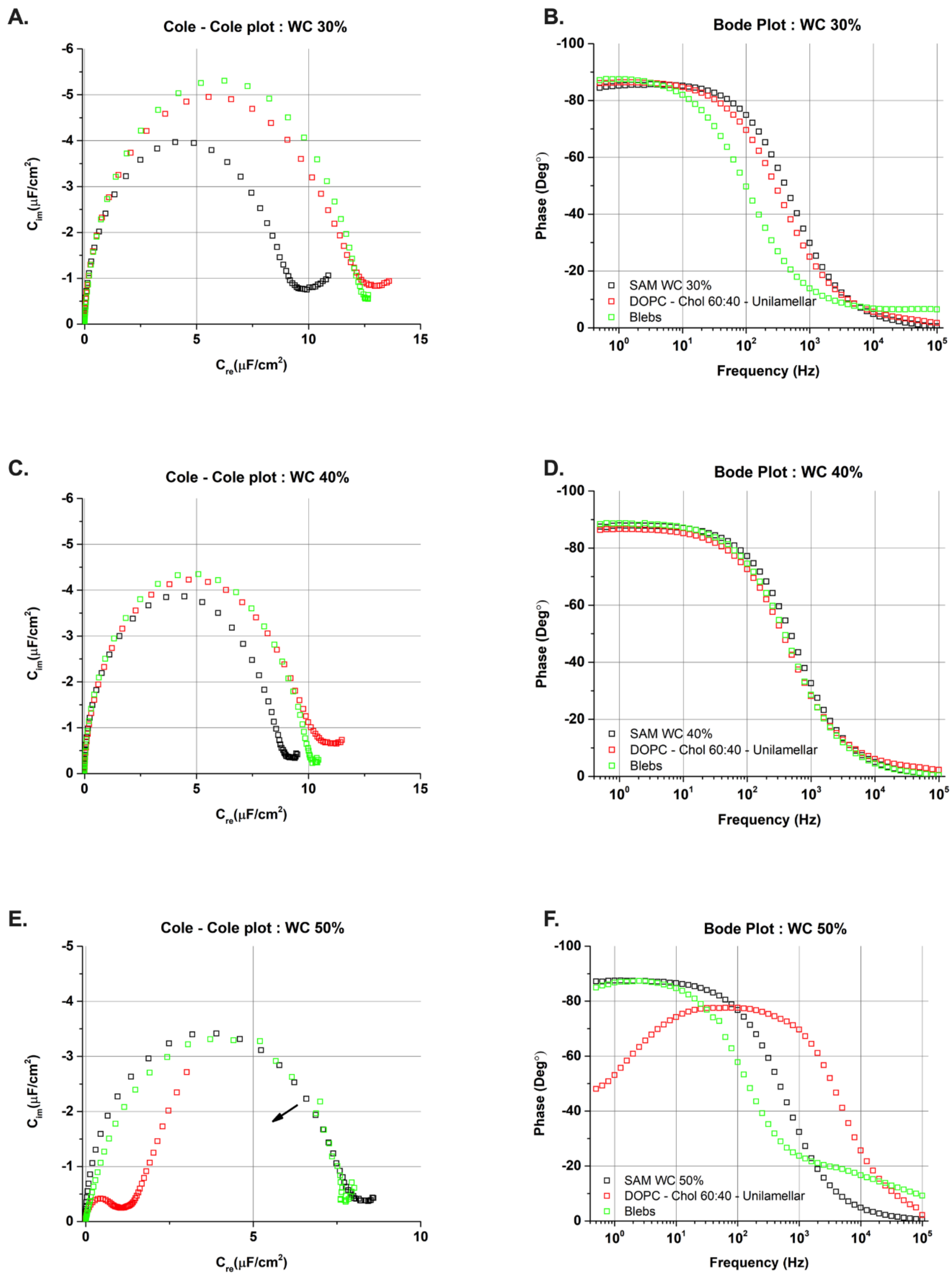

Figure S3. Electrochemical impedance spectrum of the blebs and unilamellar vesicles on increasing concentration of the anchor compounds that constitute the self-assembled monolayer(SAM) layer. The corresponding Cole-Cole(A,C&E) and Bode(B,D&F) plots are shown for the SAM layer containing 30% WC (A & B), 40% WC (C & D) and 50% WC (E &

F) respectively. Unilamellar vesicles formed using 50% WC resulted in the formation of tBLMs as inferred from the Cole-Cole plot by the decrease in the diameter semi-circular feature (arrow) between the SAM spectrum of the unilamellar vesicles (E).

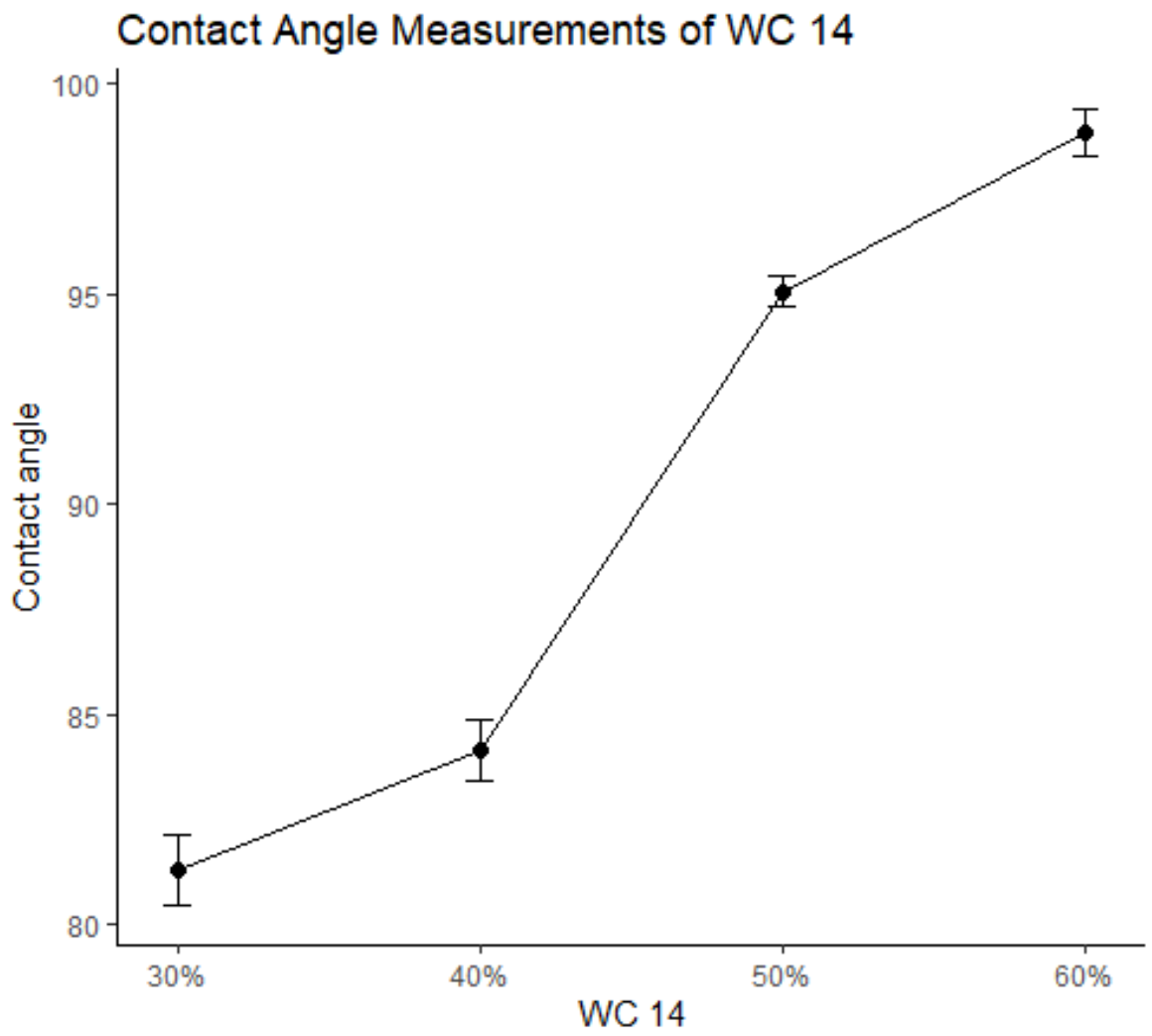

Figure S4. Contact angle measurement plot showing the increasing contact angle values for increasing WC. Line joining the points is used for visual representation only.

Table S1. Temporal evolution of the specific capacitance (µF/cm^2) and admittance (µS/cm^2) for tBLMs derived from blebs and unilamellar vesicles over a 6-day period

	Capacitance (µF/cm^2)		Admittance (µS/cm^2)	
	Blebs	Unilamellar	Blebs	Unilamellar
1 h	0.87 ± 0.16	0.78 ± 0.04	43.6 ± 27.7	5.1 ± 1.0
1 day	0.88 ± 0.20	0.79 ± 0.04	33.8 ± 23.1	6.7 ± 0.5
6 days	0.95 ± 0.14	0.80 ± 0.04	36.6 ± 27.8	21.2 ± 6.4

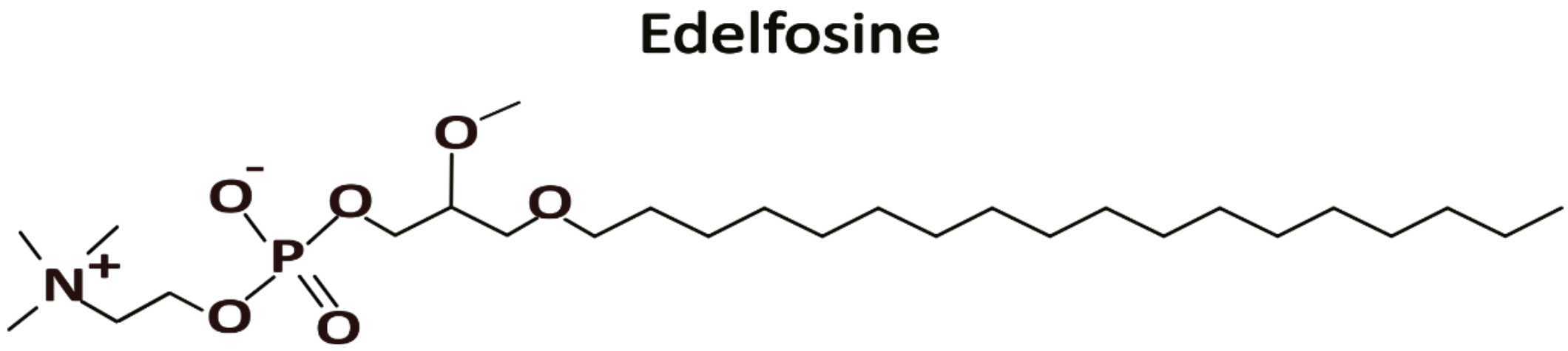

Figure S5. Structure of the anti-cancer agent Edelfosine.

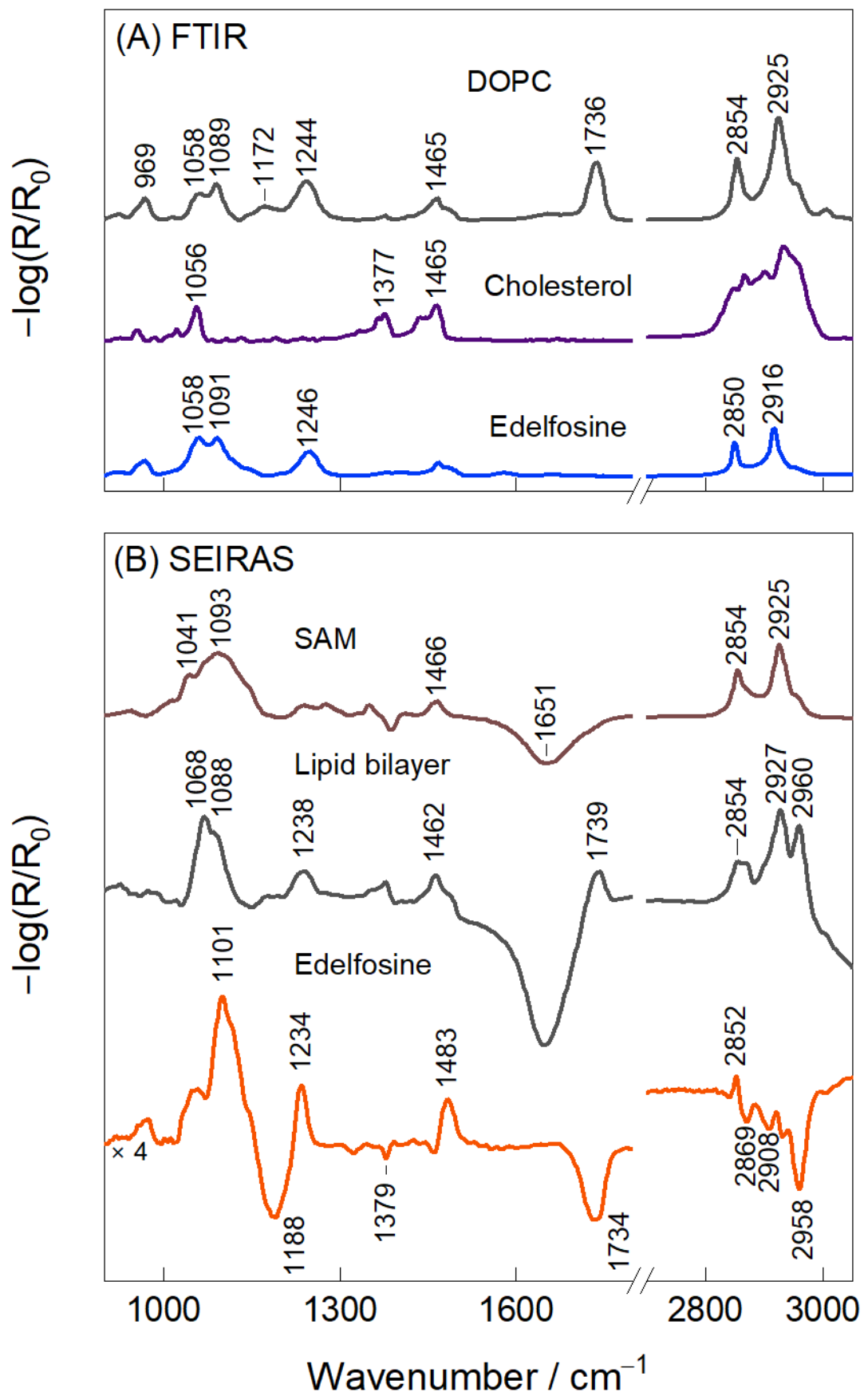

Figure S6. (A) Transmission FTIR spectra of bulk DOPC, cholesterol, and edelfosine deposited on a CaF_2 window. (B) SEIRAS spectra of a SAM (30:70 mol% WC14:ME), a lipid bilayer (60:40 mol% DOPC:cholesterol), and edelfosine at 60 min after its injection into a measurement cell. The SAM spectrum was referenced against a clean Si surface in water; the tBLM spectrum was referenced against the SAM in water; the edelfosine spectrum was referenced against the tBLM in water.

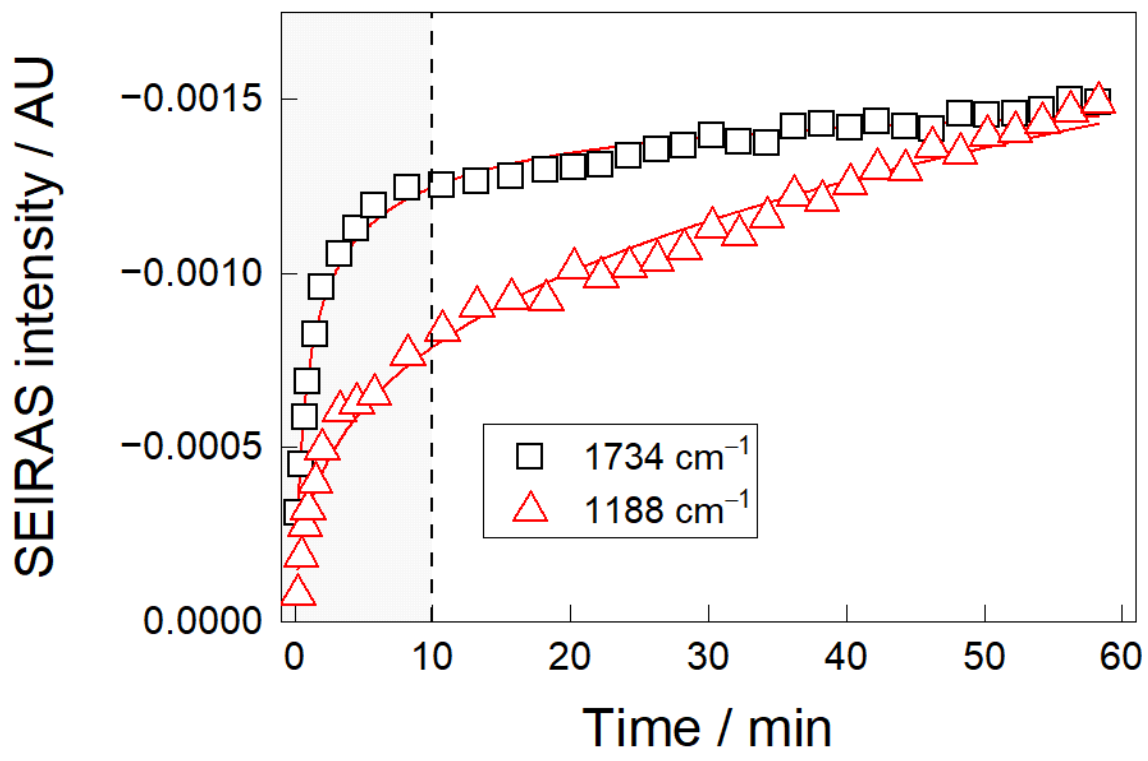

Figure S7. Temporal evolution of the ν(C=O) band intensity at 1734 cm^{-1} and ν(C–O) intensity at 1188 cm^{-1} of DOPC in the tBLM following edelfosine injection into the measurement cell.
